# Supplementary material for: Towards FAIR protocols and workflows: the OpenPREDICT use case
Source: PeerJ Comput Sci. 2020 Sep 21;6:e281. doi: 10.7717/peerj-cs.281 (PMC7924452; doi:10.7717/peerj-cs.281)
Supplement: Supplemental Information 1 [file peerj-cs-06-281-s001.pdf]

FAIR is as FAIR does:
Integrating data publishing principles in scientific workflows

NWO / eScience Center Project 628.011.011 / NLeSC P 17.0201

Deliverable 1.1: Report on Interviews and Requirements

24 January 2019

Authors:
Remzi Celebi, Ahmed Hassan, Harald Schmidt, Roel Zinkstok, João Moreira, Lars Ridder, Valentina Maccatrozzo, Tobias Kuhn, Michel Dumontier

Goal of the interviews

We conducted structured interviews with prospective users of our framework to formulate relevant user requirements. Those user requirements are essential to infer the design and implementation requirements of the framework. The interviewees were introduced briefly to the idea of the framework, as well as the core ideas behind FAIR principles. The main goal of these short interviews was to align the planned functions of the framework with the needs of the researchers, especially from the drug discovery and repurposing domains.

Background of interviewees

We interviewed 6 researchers with different backgrounds, and mostly working within the field of drug discovery and repurposing from different perspectives ranging from preclinical research to computational biology. They use different approaches such as network modeling, data integration, and image processing. They mostly use Python and R as programming languages for their work.

Interview Structure

We conducted the interviews both in person and via teleconference. The interview started by explaining the nature of the project and its goals, followed by the interviewees quickly introducing themselves, and finally the main questions. The questions were asked one by one, and follow-ups or clarifications were also included after the interviewees' answers. The questions were asked by one interviewer while the other person acted as a scribe for the conversation. The interviews were scheduled for 30 minutes and they ranged between 20 to 45 minutes. Listed below are the main questions:

1. What is the nature of your work?
2. How do you create or use datasets/workflows/protocols? Provide an example.
3. What challenges do you face when you do develop, implement, reuse, or reproduce a workflow? Do you have an experience or a story regarding these challenges?
4. Where could there be efficiency be gained?
5. What programming languages do you use in your work?
6. How could your projects/workflows be easier to find, access, interoperable, and reusable (FAIR)?

Datasets and workflows

We listed the datasets and workflows (steps) that are commonly used by these researchers based on their answers.

- Datasets

**Data source**	**Content**
Gene Expression Omnibus (GEO)	Gene expression
STRING	Protein-protein interactions
HMDB	Metabolomics
Drugbank	Drug, target, chemical structures and etc.
ChEMBL	Drug-like molecules, targets, assays
ConnectivityMap	Drug assays and drug-gene profiles
KEGG	Pathway

- Workflows

**Described Workflow/Workflow step**	**Ontology references**
Query repositories	http://edamontology.org/operation_0224
Parsing and mapping data	http://edamontology.org/operation_1812 http://edamontology.org/operation_2429
Clean data	http://edamontology.org/operation_2409 (not exact term)
Merge/aggregation	http://edamontology.org/operation_3436
Network modeling and simulation	http://edamontology.org/operation_3562
Similarity calculation	http://purl.obolibrary.org/obo/OBI_0200113
Hyperparameter optimization	https://www.w3.org/ns/mls#HyperParameterSetting
Model Evaluation	https://www.w3.org/ns/mls#ModelEvaluation
Western Blot	http://purl.obolibrary.org/obo/OBI_0000854
GO Enrichment analysis	http://edamontology.org/operation_3501

Network visualization	http://edamontology.org/operation_3083

User stories

The interviewees shared their experiences with us, and these experiences were mapped to the user stories. These user stories are high level requirements that could be translated later into the technical requirements for the framework.

- As a researcher, I can share my workflows in a repository or database so that my workflows could be findable by other researchers.
- As a researcher, I need a platform or workbench to reproduce my experiments so that I can re-run the experiments and reproduce the results.
- As a researcher, I can access the code and the data related to the workflow so that I can understand and improve the workflow or the method.
- As a researcher, I need every detail of the computational and manual workflow of a study to be clearly defined and documented so I can reproduce the study.
- As a researcher, I can use community standards and tools so that I can share my workflow with others easily.
- As a researcher, I can search for existing datasets based on keywords, concepts (e.g. a particular drug) and metadata (e.g. date or license) so that I can find potentially useful datasets.
- As a researcher, I can keep track of how the results of the workflow were generated with their provenance so I can perform a systematic analysis.
- As a researcher, I can query historical versions of workflows and their metadata so that I can understand the evolution of the workflow as well as debug problems.
- As a researcher, I can save the meta-parameters of my workflow steps separately so that I can easily share and prompt reuse of the workflow as a template with the meta-parameters as metadata to specific runs.

General Findings

The interviewees stated that they experience many challenges in reproducing their or others' work. These challenges include:

- The paper might not include many details such as workflow steps, data cleaning and filtering.

- The dataset could be not provided or is not accessible anymore. In addition to this, the information such as parameters, design details used in reproducing the results could be missing.
- Recording of manual processes or workflows is usually missing or incomplete.
- The results or hypotheses could change due to the update of the data. When they cannot reproduce a workflow or do not understand it enough, the researchers try to contact authors, but the rate of response to their questions is very low.
- Often, software libraries, packages and tools version used are not explicitly recorded, besides the fact that they might not be maintained or updated with no access to previous releases used in the original workflow.

Most of the answers show that there is no one-size-fits-all solution to these challenges, nevertheless we collected several suggestions to address the aforementioned challenges:

- Define at least a generic diagram of each computational and manual workflow.
- Make the dataset, or at least the metadata of the dataset, accessible. Also, adding provenance and using versioning for data would be useful.
- Use community standards and tools such as GitHub or figshare.
- Package the code with its dependencies, for example using docker.
- Separate the workflow steps from the workflow hyper-parameters, so that one can run the same workflow many times without changing the workflow itself.

Conclusion

Based on the feedback from the interviewees, we propose some essential requirements for our framework. As a first step, the FAIRness of datasets should be evaluated. Some of the most commonly used datasets are already provided as FAIR data in the bio2rdf project. For the remaining data, a FAIRfication process should be devised by the researchers. Second, the benchmark should allow workflows to be defined in programming languages and supporting semantic annotation of computational workflows. The researchers also indicated that they generally use R and Python. Hence, the third requirement is wide programming language support to make it easier for the community to adopt such a benchmark. Finally, workflow should be stored in a repository maintained by a community or our team. The workflows should also be defined such that the abstract and implementation levels (i.e., parameterisation details) are separated so that the same workflow can be run repeatedly without changes to the abstract definition. Experimental Standard Operation Procedure (SOP) documents are in plain English, it might be a good start to add them as manual workflows.

Appendix 1: Survey responses

Questions	Responses
Main field of work	1-Computational systems medicine<br>2- Pharmacology, experimental<br>3- data science<br>4- Computational modelling<br>5- Bioinformatics<br>6- Computer Science
What is the nature of your work?	1- Biomedical data retrieval from public databases<br>- Data Integration on graph-based schemas (parsing, mapping and schema definition)<br>- Multidimensional/multilayer network construction and analysis (mostly statistical and clustering)<br>- Disease biomarkers prediction from networks using machine learning<br>- Network enrichment analysis (gene expression, GO terms, etc...)<br><br>2- Preclinical research, working with animals or cells. doing molecular biology assays<br><br>3- computational drug discovery using machine learning models<br><br>4- Modelling and simulation of biological data, signaling pathways, networks, aka system biology<br><br>5- Network based modelling. Integrating data on diseases, drugs and PPIs and use this information for drug repurposing, relations between diseases-diseases, diseases-drugs, etc...

	6- Data informatics/science in the field of bioinformatics. Working on image recognition on echo imaging to find max ejection fraction. Working on MarketScan database, for analysis of medical and pharmacological correlations for diseases and drugs.
How do you create or use datasets/workflows/protocols? Please provide an example.	1- # Datasets:<br>- Combining public datasets (ex: PPIs, protein-metabolite, gene-disease, etc...)<br>- Using data generated from wet-lab experiments in our lab<br><br># Workflows:<br>- Reusing others workflows (computational) and modifying them<br>- Creating de-novo workflows for our use cases<br>- No workflow frameworks used, but sometimes using docker<br>- Using Jupyter notebooks or normal scripts<br><br>2- Already defined protocols, used with optimisation, ex: Western blot protocol<br>The optimisation is for the protein and antibody concentrations, to get a good output signal<br>Mostly protocols from publications, and few changes. They are extracted from the method section of publications manually. Not stored in a structure form.<br><br>3- Use SPARQL to query repositories such as Drugbank, KEGG, PharmGKB and NDF-RT or download raw data and parse and clean, merge data with Python Pandas library<br>-Create workflows in Jupyter Notebook<br><br>4- Use SPARQL to query repositories such as Drugbank, KEGG, PharmGKB and NDF-RT or download raw data and parse and clean, merge data with Python Pandas library<br>-Create workflows in Jupyter Notebook<br><br>5- # Data: ex: transcriptomics data, GEO, ArrayExpress, ExpressionAtlas<br># Workflows:<br>Two general workflows:<br>One:<br>- Cleaning

	- Finding causalities and relations<br>- Verify generated networks (via models)<br>- Simulating the networks, to generate predictions<br><br>Two:<br>- Go to literature for pathways (manual or mining)<br>- Expanding or combining the pathways<br>- Convert to network<br>- Verify<br>- Simulate and predict<br><br># Datasets<br>mostly public databases: NCBI, BIANA, DrugBank, Chembl, GEO, from articles<br><br># Workflow<br>Pseudo workflow: (python structure)<br>- Parameters in config file<br>- Main.py file for main code<br>- Other files<br>For some project, using Jupyter notebooks, for sharing and collaboration<br><br>6- # Databases:<br>Mostly public databases: MarketScan, google datasets search<br><br># Workflow:<br>Not a specific workflow. Depends on the data. Using steps of analysis from other research. Using discovery methodology to explore the data and what kind of analysis is possible with it. Discovery, preprocessing, analysis, visualization
What challenges do you face when you do develop, implement, reuse, or reproduce a workflow? Do you have an experience or a story regarding these challenges?	1- Finding and querying related works (How not to miss related work)<br>- Extracting methods (exact steps) from papers (if no code provided)<br>- Finding data or alternatives sources when datasets are not provided or proprietary<br><br>Story/experience:<br>I had a task to update the human disease network, which is build via gene-disease associations. The original paper used a database that still exists, but the old retrieved data was from 2006, and it needed to be up to date. The main problems I faced were:

	- Reconstructing the steps they used to create the original network, as there were many arbitrary decisions taken to include or exclude associations<br>- When reconstructing the new network, the old decisions that were taken based on the old data were invalid for the up-to-date data. The output network didn't have the same properties as the old one. Since some of the decisions had no justification; adjusting them for the new network proved to be very challenging.<br>Some steps are not mentioned, or ignored. You have to contact the researchers to get details which is very time consuming, or try different values for the optimisations or methods until getting results.<br><br>The storing of the protocols is done as SOPs in a standard template from the lab (word doc), and stored in google drive for the lab team to use<br><br>Story:<br>Doing enzyme activity assay, and it had no protocol. He got the information from a publication, and they faced a problem because the PH levels for the buffer was not mentioned. They had to try to go to different papers and try different PH levels to be able to reproduce the original results<br><br>2- Some steps are not mentioned, or ignored. You have to contact the researchers to get details which is very time consuming, or try different values for the optimisations or methods until getting results.<br><br>The storing of the protocols is done as SOPs in a standard template from the lab (word doc), and stored in google drive for the lab team to use<br><br>Story:

	Doing enzyme activity assay, and it had no protocol. He got the information from a publication, and they faced a problem because the PH levels for the buffer was not mentioned. They had to try to go to different papers and try different PH levels to be able to reproduce the original results<br><br>3- Challenges: frequent change in datasets, accessibility of data and code<br>Missing data, feature in original dataset, lack of given details (eg. parameters, design) in the study<br>An experience: I tried to reproduce the PREDICT study for drug repurposing, and faced many challenges during reproducing the study. First, the datasets used for creating the features were not provided within the study. I had to construct the features using open, accessible datasets such as DrugBank and KEGG and SIDER. Another challenge is the integration of the datasets, each dataset use different naming for drugs and diseases and luckily bio2rdf project helped me a lot to overcome to mapping and matching the drugs and diseases across different datasets. But I had to find additional mapping for drugs in SIDER. The authors did not mention which version of the library they used for creating semantic similarity feature. Some implementation details such as calculation of combined features are not clear and there was no the code provided to refer.<br><br>4- Challenges:<br>- Finding same datasets<br>- Reconstructing the same work first, before repurposing<br>- The datasets are not open, or parts of it<br>- papers written by statistician, different perspective of what is needed for reproducibility, dropping information such as which specific parameters for algorithms

	- 50% response rate of paper authors<br>- Library and software gets updated and that makes problems for rerunning workflows<br><br>Story:<br>He used HighTech tool from 1998. Needed to focus on the time aspect of the cell processes. New softwares lack some features of that tool. The old tool was implemented for archaic Linux, and couldn't update since it relies on older libraries.<br><br>5- The approach is very dynamic, data change, direction of analysis change. Lots of experimentation, not big picture from the beginning.<br>- So many parameters to tweak<br>- No good planning of analysis in advance<br>Example:<br>Parse to parse DrugBank, and then something in the DB change, and then not discovering the bug until<br><br>Story:<br>Uniprot ID are dynamic and tend to change. The problem is in 2018, when you go back to some old Uniprot, they have changed. And throughout time, what you see might not stay the say. They don't include historical record. So there is a danger that proteins are not exactly what they are the same. Also for DrugBank, if you want an updated dataset. When I run my existing script, it fails due changed in format or so.<br><br>6- Data referenced was raw data, but current link refers to transformed data<br>- No explanation of changes and data transformation<br>- Documentation of steps, data cleaning, filtering, selections, etc...

Where could there be efficiency be gained in your workflows?	1- Being able to reproduce and compare other approaches/workflows easily (i.e. with little time and adaptations as possible)<br>- Automate the rerunning of workflows when doing iterative development of the approach<br><br>2- It will save time for example for non commercially available compounds as it cuts the need to re-optimise the protocol again<br>- Easily attach the meta-data of the experiments to the protocols<br>- Record all the experimental steps instead of keeping them hidden on publication<br>- Automate the process of running the protocols<br>- Facilitate the search for protocols and with provenance and meta-data you can get all the details of a specific run and optimisations<br><br>3- Make it easier to do a comparative study if the reproducible workflows are available.<br><br>4- The interlinking of resources and codes<br>- Getting a pipeline made of different tools to work<br>- Much of the documentation is missing, which requires going through the whole workflow<br>- Licensing, not very clear what type of license is used<br><br>5- Save time especially with large scale datasets<br>- Rely on some available workflow<br>- When you want to share, it would answer the demand of journals and other researchers.<br>- Tradeoff between the effort to use the framework, and how much time and effort it saves<br><br>6- Easier experimentation of different workflows, and validation of old results<br>- Easier modifications and incremental improvement

What programming languages do you use in your work, if any?	1- Python, R, Bash<br>2- None<br>3- Python, Java<br>4- R, Python<br>5- Python for analysis<br>R for visualization<br>6- Applications: Java, C#, Angular<br>Research: Python, R, Matlab
How could your projects/workflows be easier to find, access, interoperate, and reuse (FAIR)?	1- Share it in dedicated workflow databases<br>- Provide links to the data source<br>- Force or at least incentivise describing all steps and their rationale<br>- Use generic and community standard tools as much as possible<br>- Write manuals for developed tools and video tutorials<br><br>2- Create a community platform where you can ask researchers (ex: researchgate)<br>- Use of videos to explain protocols (ex: like the JOVE journal)<br><br>3-Share the workflow in an open repository<br>Provide the code and data<br>Provide manual and implementation details for your workflow/software<br>Provide a platform or tool that the researcher can re-run the experiments and reproduce the results (such as Google Colaboratory)<br><br>4- Publish in open access journals<br>- Generic diagrams of workflows<br>- Tutorial videos/animations<br>- Markdown/notebook integrated with diagrams (ex: Jupyter)<br>- Docker images

	5- Nextflow initiative: some integration with docker<br>- Wok: another workflow manager<br>- Use github or figshare, zendo<br>- Getting DOI for datasets<br><br>6- Good documentation<br>- Code in github, etc... (at least for data preparation)<br>- Explain to details all the steps, parameters, etc...
